# Chronic active Epstein–Barr virus infection as the initial symptom in a Janus kinase 3 deficiency child: Case report and literature review

**DOI:** 10.1097/MD.0000000000007989

**Published:** 2017-10-20

**Authors:** Linqing Zhong, Wei Wang, Mingsheng Ma, Lijuan Gou, Xiaoyan Tang, Hongmei Song

**Affiliations:** Department of Pediatrics, Peking Union Medical College Hospital, Chinese Academy of Medical Sciences and Peking Union Medical College, Beijing, China.

**Keywords:** chronic active Epstein–Barr virus infection, Janus kinase 3, primary immunodeficiency

## Abstract

**Rationale:**

With the progress of sequencing technology, an increasing number of atypical primary immunodeficiency (PID) patients have been discovered, including Janus kinase 3 (*JAK3*) gene deficiency.

**Patient concerns:**

We report a patient who presented with chronic active Epstein–Barr virus (CAEBV) infection but responded poorly to treatment with ganciclovir.

**Diagnoses:**

Next-generation sequencing (NGS) was performed, including all known PID genes, after which Sanger sequencing was performed to verify the results. Genetic analysis revealed that our patient had 2 novel compound heterozygous mutations of *JAK3*, a gene previously reported to cause a rare form of autosomal recessive severe combined immunodeficiency with recurrent infections. The p.H27Q mutation came from his father, while p. R222H from his mother. Thus, his diagnosis was corrected for JAK3-deficiency PID and CAEBV.

**Interventions:**

Maintenance treatment of subcutaneous injection of recombinant human interferon α-2a was given to our patient with 2 MU, 3 times a week.

**Outcomes:**

Interferon alpha was applied and the EBV infection was gradually controlled and his symptoms ameliorated remarkably. Our patient is in good health now and did not have relapses.

**Lessons:**

The diagnoses of PID should be taken into consideration when CAEBV patients respond poorly to conventional treatments. Good results of our patient indicate that interferon α-2a may be an alternative treatment for those who are unwilling to accept hematopoietic stem cell transplantation (HSCT) like our patient. Literature review identified 59 additional cases of JAK3 deficiency with various infections.

## Abstract 2

Supplemental Digital Content is available in the text

## 1 Introduction

With the progress of sequencing technology, an increasing number of atypical primary immunodeficiency (PID) patients have been discovered, including Janus kinase 3 (JAK3) deficiency. *JAK3* gene mutations have been previously described to cause a rare form of autosomal recessive severe combined immunodeficiency (SCID) with severely deficient function of the immune system. JAK3 is required for signaling of cytokine receptors that employ the common gamma chain (γc).^[1]^ The phenotypes of JAK3-deficiency are variable and complex, ranging from symptomless to severe and recurrent infections. Its typical clinical manifestations include early-onset and recurrent infections, absence of T and natural killer (NK) cells but normal number of less functional B cells in peripheral blood. Herein, we report an atypical JAK3 deficiency patient who presented with chronic active Epstein–Barr virus (CAEBV) infection and was subsequently identified to possess 2 compound heterozygous JAK3 mutations. The literature review describes genotypes, phenotypes, and therapies of JAK3-deficiency.

## 2 Case report

A 12-year-old boy presented with recurrent fever for more than 2 years and was referred to Peking Union Medical College Hospital in 2014. The recurrent fever began without obvious predisposition and lasted 4 to 14 days per attack. The intervals between episodes range from 4 to 20 days. Temperature peak was up to 40°C. No cough, expectoration, rash, joint pain, or other discomfort appeared. He was referred to local clinics and had been diagnosed with Epstein–Barr virus (EBV) infection many times with positive EBV antibodies (EBV-Ab) and elevated plasma virus copies. Details of his physical examination, laboratory results, and therapies were unavailable.

He visited a hospital in Beijing owing to another fever in February 2014. Enlargement of lymph nodes and hepatosplenomegaly were discovered in physical examination. Assay of EBV-Ab and virus copies were performed, which indicated that IgA/early antigen (EA), IgA/viral capsid antigen (VCA), IgG/VCA, NA-IgG were positive and the virus copies were elevated to 3.45 × 10^5/mL. Diagnosis of EBV infection was made and then ganciclovir was employed, which ameliorated his symptoms and reduced the virus copies.

In May 2014, his fever relapsed and then he was referred to Peking Union Medical College Hospital for further treatment. Reviewing the history of the patient before the onset of recurrent fever, we found that he had frequent respiratory infections with twice a month. Physical examination indicated that his growth and development were within normal limits, weighing 38.5 kg and 146 cm in height (both in the range of the tenth percentile to the twenty-fifth percentile). Ichthyosis rash could be seen on his lower limbs. Several enlarged lymph nodes were palpable in the anterior region of the neck. No erythema or exudate of the throat or enlargement of tonsils was observed. There were no obvious abnormalities with cardiopulmonary examination. On the abdominal physical examination, hepatomegaly was found with 2 cm below the ribs, medial hardness, and a clear margin. Spleen could not be palpated. No deformity of joints was found as well.

**Figure 1:**
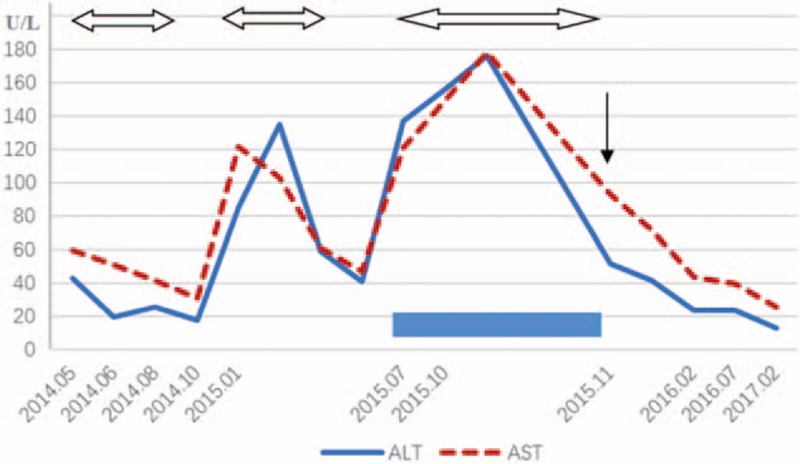
The level of ALT and AST changing with time (U/L). The double arrow means treating with ganciclovir, rectangle means cyclosporine, and single arrow means interferon.

**Figure 2:**
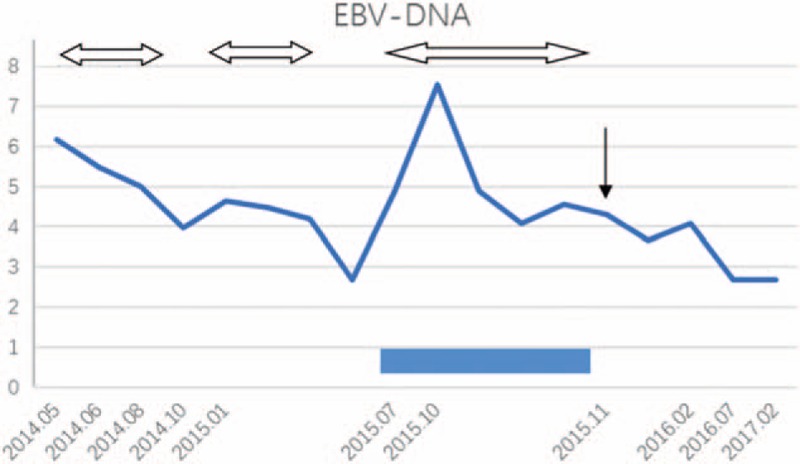
The virus copies varied with time. Logarithmic function was used to transform the EBV copies. The double arrow means treating with ganciclovir, rectangle means cyclosporine, and single arrow means interferon.

On laboratory examination, peripheral blood count showed a decreased number of neutrophils and proportions of lymphocytes and neutrophils were inverse (63.2% and 25.9%, respectively). Acute phase reactants increased (erythrocyte sedimentation rate was 34 mm/h and C reactive protein was 15 mg/L). Liver function tests revealed slightly elevated glutamic oxaloacetic transaminase (AST) and normal alanine aminotransferase (ALT) (see Fig. 1). Serum immunoglobulin was normal (IgG, 16.44 g/L; IgA, 5.24 g/L; IgM, 0.75 g/L) with decreased T cells in the peripheral blood (see Table 1). Table 1 summarizes the values of lymphocyte subsets changing with time and therapy. The numbers of CD3+ T cell especially the CD4^+^ T cell markedly decreased, while NK cell significantly increased. These abnormal results persisted despite treatment with cyclosporine A for 4 months from July 2015 to November 2015. Recombinant human interferon α-2a began to be used in November 2015 with a dose of 1 MU, 3 times a week. Four months later in February 2016, the above abnormality still existed despite the remission of his symptoms and improvement of other laboratory examinations. Dosage of recombinant human interferon α-2a was then added to 2 MU, 3 times a week. Henceforward, the values of lymphocyte subsets gradually returned to normal level. Positivity of EBV-Ab (see Table 2) and elevated virus copies (1.5 × 10^6 copies/mL, see Fig. 2) were observed. As we can see in Table 2, IgA/EA, IgA/VCA, IgG/VCA, NA-IgG were persistently positive no matter what treatment was used. Abdominal ultrasonography indicated hepatosplenomegaly (oblique diameter of right lobe of liver was 13.0 cm and spleen diameter was 14.3 cm). Cardiac ultrasound showed that he had patent ductus arteriosus.

**Table 1:**
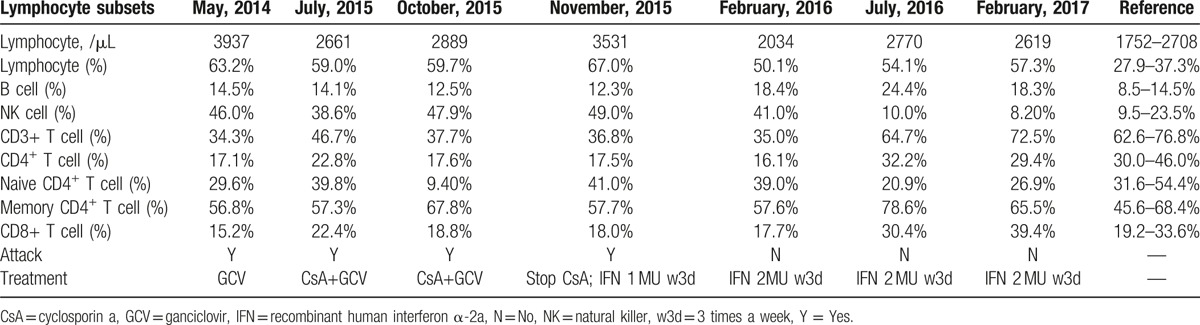
Values of lymphocyte subsets.

**Table 2:**
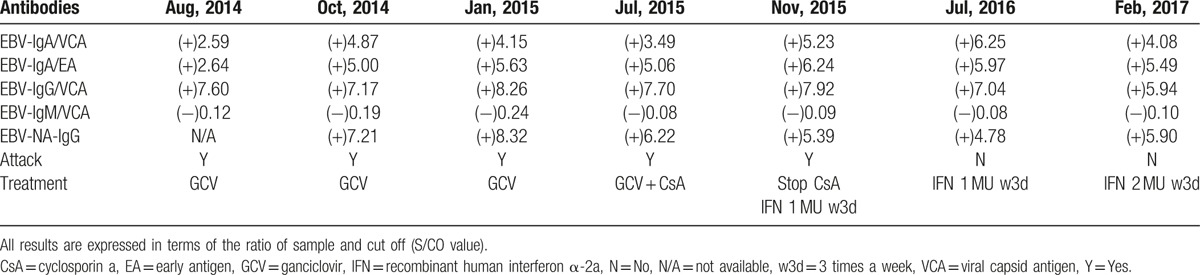
The result of Epstein–Barr virus antibodies.

His symptoms, abnormal signs, and laboratory indexes repeatedly occurred during the follow-up, which were improved with repeated treatment of ganciclovir. The diagnosis of CAEBV was established on the basis of diagnostics guideline proposed by Okano et al.^[2]^ The following aspects of our patient fulfilled the Okano guideline: recurrent infectious mononucleosis-like symptom include fever, swelling of lymph nodes, and hepatosplenomegaly; unusual pattern of anti-EBV antibodies with raised anti-VCA and anti-EA, and detection of increased EBV genomes in the peripheral blood; and exclusion of other chronic illness such as other infections, autoimmune diseases, and neoplastic diseases upon completion of related examinations.

**Figure 3:**
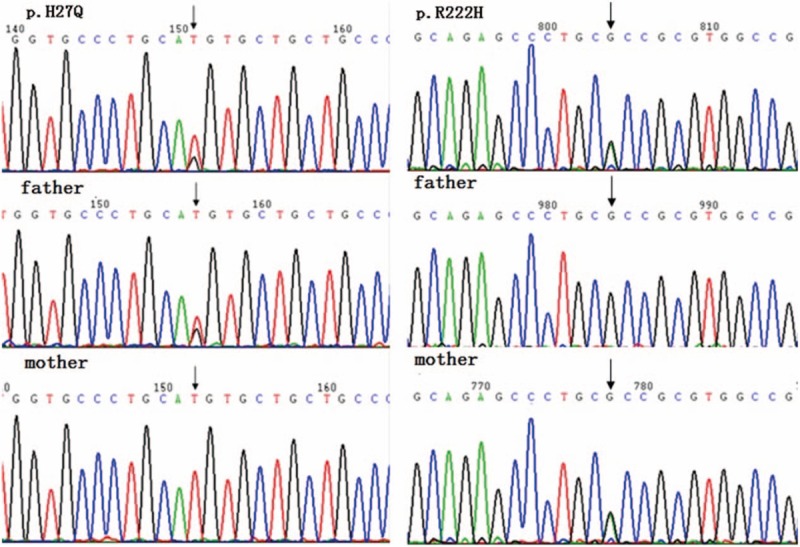
The Sanger sequencing revealed novel compound heterozygous mutations of JAK3. The c.81T>G, p.H27Q mutation in exon 2 came from his father, while c.665G>A, p. R222H in exon 6 from his mother.

The repeated use of ganciclovir could not prevent the frequent relapses of EBV infection for our patient. The combination of cyclosporine A for 4 months could not reduce the virus copies to normal level as well. With the possibility of immunodeficiency disease, next-generation sequencing (NGS) was performed to analyze all known PID genes followed by Sanger sequencing to verify results. The patient possessed 2 novel compound heterozygous mutations of JAK3 (see Fig. 3). The p.H27Q mutation came from his father, while p. R222H from his mother. Thus, his diagnosis was corrected for JAK3-deficiency PID and CAEBV. Antiviral therapy was replaced with subcutaneous injection of recombinant human interferon α-2a with 1 to 2 MU, 3 times a week since November 2015. The virus copies gradually decreased to the normal level and his symptoms ameliorated significantly. Moreover, ALT decreased to normal level after 3 months. His latest follow-up was in February 2017. He has hardly had a fever for nearly 1 year and the virus copies and ALT were normal.

## 3 Methods

The method of NGS refers to Xiao Zhang's study^[3]^ and the list of the genes captured in the present study can be found in Table, Supplemental Digital Content 1. A literature search was conducted in PubMed with search term JAK3 deficiency. Additional search came from the references of identified literature. Demographic and clinical data were summarized, including ethnicity, parental consanguinity, clinical manifestations, immunological phenotype, immunoglobulin level, and treatments.

Written informed consent was obtained from our patient and his parents, and the study was approved by the Ethics Committee of the Peking Union Medical College Hospital.

### 3.1 Review of JAK3 deficiencies and discussion

#### 3.1.1 Review of JAK3 deficiencies

The clinical manifestations of JAK3-deficiency are variable and complex, ranging from symptomless to severe and recurrent infections. Early-onset is a traditional feature of JAK3-deficiency and most patients presented with symptoms within the first few months after birth. The second characteristic is recurrent infections, including bacterial, viral, and fungal infections involving the respiratory tract, digestive tract, urinary system, central nervous system, and skin. Opportunistic infections and deep infections involving the liver and bone marrow have also been reported.^[4–6]^ In addition, disseminated infection after BCG and varicella vaccine inoculation have occurred.^[6,7]^ A majority of patients developed growth retardation. Some patients came from families with a medical history of PID and consanguineous marriage.^[4–17]^ Other atypical symptoms such as skin warts^[8]^ and extensive granuloma ^[15]^ have been found. Some patients are almost normal.^[4,7,8,12–14,16,18,19]^

The immunological phenotypes of those previously reported cases were usually T-B+NK− (marked reduction in T cells and NK cells with preservation of B cells), and only few patients were T-B+NK+ (marked reduction in T cells with preservation of B cells and NK cells).^[12,14,16,17]^ Lacking assistance from T-cell cytokines, the development and function of B cells are abnormal and thus immunoglobulin is reduced.^[18,20–22]^

The human *JAK3* gene contains 23 exons and spans 3375 base pairs, translating into an 1124-amino acid protein. JAK3 contains 7 homology domains, namely JH1 to JH7. The C-terminal JH1 domain has catalytic activity and JH2 is a pseudo-kinase domain bearing regulatory function. The N-terminal JH7 and JH6 domain interact directly with the gamma chain of cytokine receptor.

**Figure 4:**
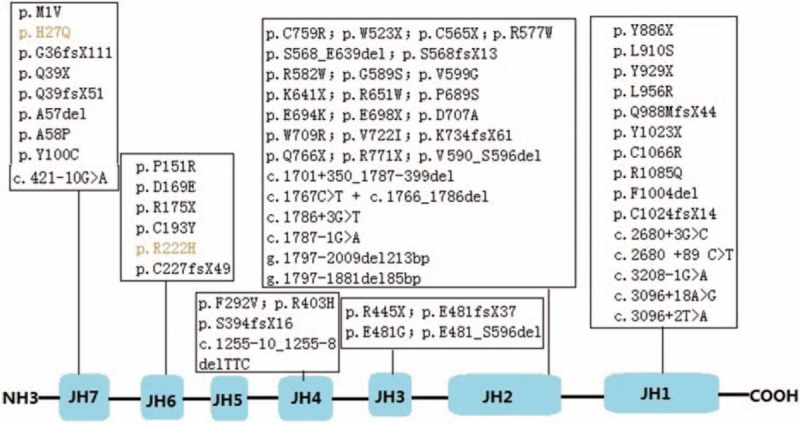
Correspondence between JAK3 mutations and its 7 domains. The c.3054 G>A^9^ was not included, as the mutation was not clearly expressed.

So far, 59 JAK3-deficiency patients have been reported. Figure 4 shows the corresponding relationship between JAK3 mutations and the 7 domains.^[4–8,10,12–20,22–32]^ As shown in the figure, mutations were mainly located in JH2 domain. The JAK3 mutations of our patient were within the JH6 and JH7 domain, disturbing the interaction with the gamma chain of the interleukin 2 receptor.

The optimal treatment for JAK3-deficiency is hematopoietic stem cell transplantation (HSCT) or bone marrow transplantation (BMT). So far, there were 32 JAK3-deficiency patients who were treated with HSCT, among whom 26 were still alive and significantly improved,^[5–7,9,11,22,25]^ 5 were deceased,^[4,22]^ and 1 was without reported outcome.^[22]^ One patient developed hemophagocytosis ^[11]^ and others with obvious rejection reaction after HSCT or BMT.^[8,15,20,31]^ Overall, the outcome was satisfactory after HSCT. Other therapies for JAK3-deficiency patients include substitution with immunoglobulin and antibiotic prophylaxis for the rest of their lives. The clinical characteristics and therapies of JAK3-deficiency are summarized in Table 3 .

**Table 3:**
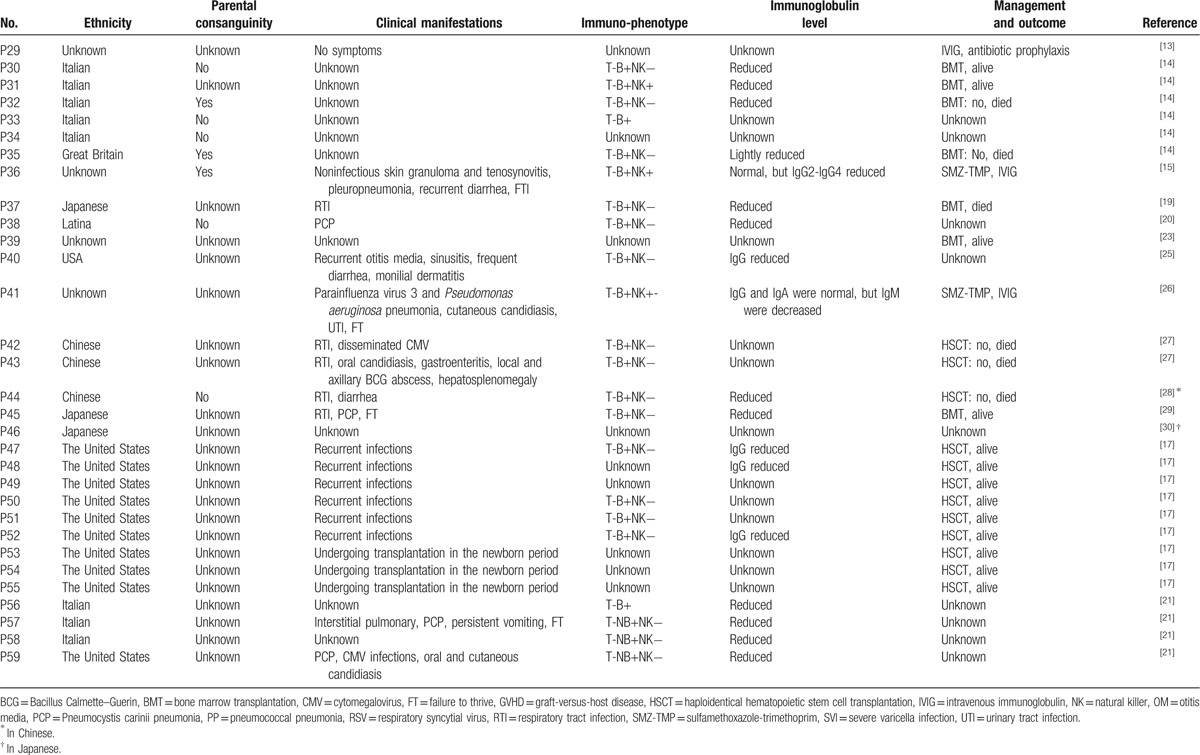
Clinical characteristics and therapies of JAK3-deficiency SCID.

**Table 3 (Continued):**
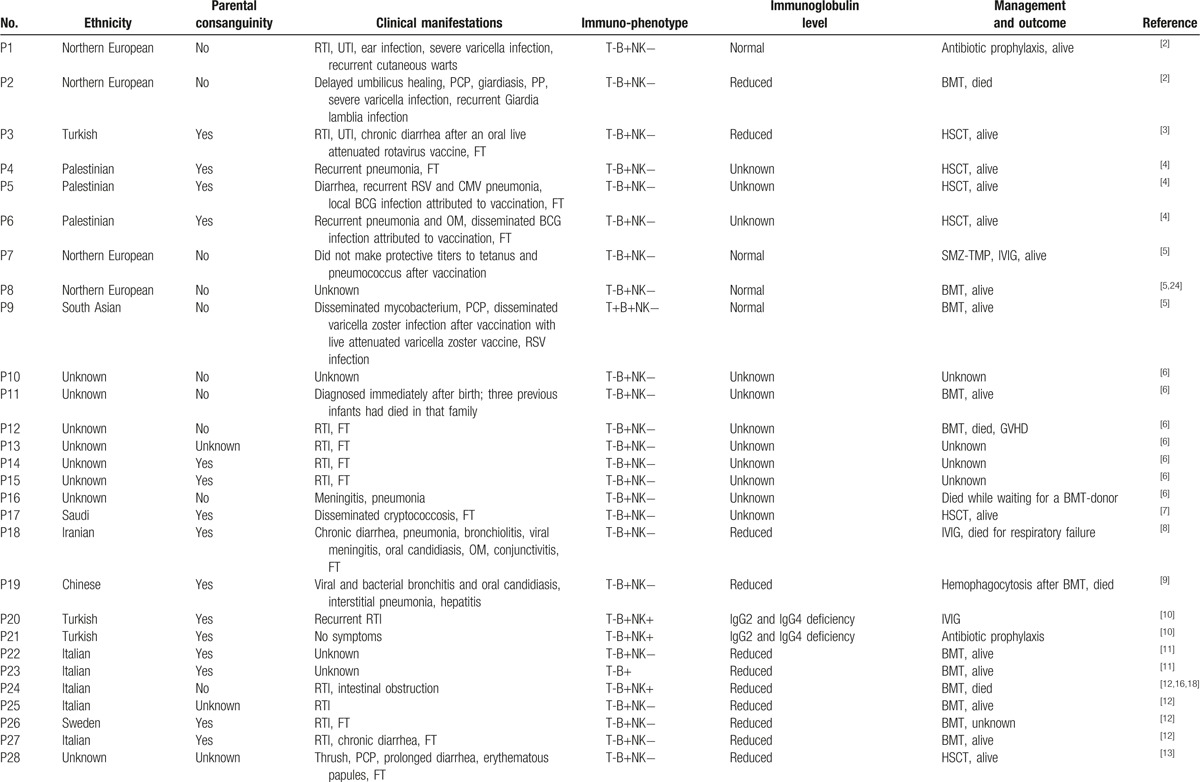
Clinical characteristics and therapies of JAK3-deficiency SCID.

## 4 Discussion

JAK3 is a tyrosine kinase belonging to the Janus kinases family, which also includes JAK1, JAK2, TYK2. Its main function is cytokine signaling. As some cytokine receptors lack enzymatic activity, they are dependent upon the intracellular JAKs to initiate signaling upon binding with their ligands (e.g., cytokines such as IL-2, IL-4, IL-7, IL-9, IL-15, and IL-21). These cytokines are crucial for the function and development of immune cells. The mutated JAK3 leads to blockage of JAK3 signal pathway, thus affecting the signal transduction of the above cytokines. As JAK3 is mainly expressed in T cells and NK cells, the common immunological phenotype of JAK3-deficiency is T-B+NK−. Although there are normal number of B cells in the peripheral blood, their function is impaired, as the above cytokines are unable to work well. As a result, both humoral immunity and cellular immunity are ultimately damaged, contributing to recurrent and various infections in patients.

The clinical manifestations of our patient are atypical with school-age onset and mild symptoms, manifesting as intermittent fever and abnormal liver function. His growth and development was not affected. What is more, his immunological phenotype was T low B+NK+, and the level of immunoglobulin was normal, which was different from the classical JAK3-deficiency. The recurrent respiratory infection in his childhood is a subtle sign to imply immunodeficiency. The atypical phenotype of our patient may be due to the possibility that these mutations lead to partial function loss of the JAK3, thus signal transduction was partly blocked. Atypical JAK3-deficiency has been described previously.^[4,7,8,12–14,16,18,19]^ Some studies suggested that specific JAK3 mutations may be hypomorphic or revertant mosaicism, thus allowing JAK3 mediated signal transduction.^[12]^ Siblings with the same JAK3 mutations may have greatly different clinical manifestations, one with typical PID manifestations, while the other nearly normal.^[8,15,16]^ Some patients with JAK3 heterozygous mutation or without a JAK3 mutation still developed the classical PID phenotype seen with JAK3 insufficiency.^[15,16,18]^

The inconformity between genotype and phenotype brings difficulties and challenges for diagnosis. It suggests that other modified genes or some environmental factors may play a certain role on the pathogenic mechanism for JAK3-deficiency.

As life-threatening infections may occur after vaccination in JAK3-deficiency patients, early diagnosis and timely BMT can remarkably improve the prognosis. It is crucial for clinicians to make an early diagnosis. Screening is an effective way to detect the potential JAK3-deficiency patients and the main method used is T-cell receptor excision circles.^[33]^ Within the past decades, the NGS technology made rapid progress, thus increasing potential PID patients came to light. Clinician could take advantage of its unprecedented throughput to find out thousands of mutations simultaneously. Once recurrent or infrequent infections happened, the diagnosis of PID should be considered and targeted sequencing may be the first choice. However, manifestations of most PIDs are similar and confusing, thus NGS may be a better option in this circumstance. Nevertheless, the NGS is time-consuming as well as costing. Newborn screening with NGS is required to those with a family history of PID.

So far, some countries or regions such as Europe, the United States, Canada, New Zealand, Israel, and Taiwan have established neonatal screening for PID.^[34–38]^ However, the mainland of China has not yet set up such screening system for PID patients. More attention is needed to pay on screening for PID patients.

EBV infection is common in children and usually results in a self-limited, transient disease and viral clearance. However, when it happens to immunocompromised individuals, it is not that simple. Some types of PID are characterized by the development of EBV-associated complications or confer predisposition to EBV infection in otherwise healthy individuals, such as X-linked inhibitor of apoptosis protein deficiency, CD27 deficiency, serine/threonine-protein kinase 4 deficiency, magnesium transporter 1 deficiency, and so on.^[39,40]^ Several case reports demonstrated that when infected with EBV, PID patients are more likely to end up in poor outcome, such as lymphoma, fulminant infectious mononucleosis, EBV-associated hemophagocytic lymphohistiocytosis, or persistent EBV viraemia.^[41–45]^ Unfortunately, acyclovir-related antiviral drugs for EBV can only inhibit replication but are insufficient to eliminate the latent infection. HSCT was believed to be effective to them, as Intan's investigation revealed a survival rate of 72% regarding IL2RG/JAK3 SCID.^[46]^ Interferon α-2a is usually used in the treatment of hepatitis viruses or malignancy for its powerful antiviral effect.^[47–49]^ However, there have been few previous publications on the use of interferon alpha-2a in CAEBV patients. In consideration of its powerful antiviral effect and our patient's personal willingness, we administered interferon α-2a to attempt to stimulate anti-EBV responses and restrict EBV replication, thus alleviating viremia. The good results of our patient indicate that interferon α-2a may be an alternative treatment for those who are unwilling to accept HSCT like our patient.

In summary, we report a patient with novel compound heterozygous JAK3 mutations but incomplete loss of immune function (T-B+NK+) who presented with CAEBV as the initial symptom. The partial loss of function in JAK3 suggested by this phenotype explains the late onset of significant disease. We summarized the genotype, phenotype, and therapies of JAK3-deficiency in this paper as well.

## Supplementary Material

Supplemental Digital Content
