# Supplementary material for: Chronic active Epstein–Barr virus infection as the initial symptom in a Janus kinase 3 deficiency child: Case report and literature review
Source: Medicine (Baltimore). 2017 Oct 20;96(42):e7989. doi: 10.1097/MD.0000000000007989 (PMC5662356; doi:10.1097/MD.0000000000007989)
Supplement: Supplemental Digital Content [file medi-96-e7989-s001.docx]

| ACP5 | CD3E | DCLRE1C | IL17RA | MYD88 | RNASEH2C | TLR3 |
| --- | --- | --- | --- | --- | --- | --- |
| ACTB | CD3G | DKC1 | IL17RC | NBN | RNF168 | TMC6 |
| ADA | CD40 | DNMT3B | IL1RN | NCF1 | RNF31 | TMC8 |
| ADAM17 | CD40LG | DOCK2 | IL21 | NCF2 | RORC | TMEM173 |
| ADAR | CD46 | DOCK8 | IL21R | NCF4 | RPSA | TNFRSF13B |
| AICDA | CD59 | ELANE | IL2RA | NFAT5 | RTEL1 | TNFRSF13C |
| AIRE | CD79A | EPG5 | IL2RG | NFKB2 | SAMHD1 | TNFRSF1A |
| AK2 | CD79B | FADD | IL36RN | NFKBIA | SBDS | TNFRSF4 |
| AP3B1 | CD81 | FAS | IL6 | NHP2 | SEMA3E | TNFSF12 |
| APOL1 | CD8A | FASLG | IL7R | NLRC4 | SERPING1 | TPP1 |
| ATM | CEBPE | FCGR3A | INO80 | NLRP12 | SH2D1A | TPP2 |
| B2M | CECR1 | FCN3 | IRAK4 | NLRP3 | SH3BP2 | TRAF3 |
| BLM | CFB | FERMT1 | IRF7 | NOD2 | SKIV2L | TRAF3IP2 |
| BLNK | CFD | FERMT3 | IRF8 | NOP10 | SLC29A3 | TREX1 |
| BLOC1S6 | CFH | FOXN1 | ISG15 | NRAS | SLC35C1 | TRNT1 |
| BTK | CFHR1 | FOXP3 | ITCH | ORAI1 | SLC37A4 | TTC37 |
| C1QA | CFHR2 | FPR1 | ITGB2 | PARN | SLC46A1 | TTC7A |
| C1QB | CFHR3 | G6PC3 | ITK | PIK3CD | SMARCAL1 | TYK2 |
| C1QC | CFHR4 | GATA2 | JAGN1 | PIK3R1 | SP110 | UNC119 |
| C1R | CFHR5 | GFI1 | JAK3 | PLCG2 | SPINK5 | UNC13D |
| C1S | CFI | GUCY2C | KRAS | PMS2 | STAT1 | UNC93B1 |
| C2 | CFP | HAX1 | LAMTOR2 | PNP | STAT2 | UNG |
| C3 | CHD7 | HPS1 | LCK | POLE | STAT3 | USB1 |
| C5 | CIITA | HPS4 | LIG4 | PRF1 | STAT5B | VPS13B |
| C6 | CLEC7A | HPS6 | LPIN2 | PRKCD | STIM1 | VPS45 |
| C7 | CLPB | ICOS | LRBA | PRKDC | STK4 | WAS |
| C8A | COL7A1 | IFIH1 | LYST | PSMB8 | STX11 | WIPF1 |
| C8B | COLEC11 | IFNG | MAGT1 | PSTPIP1 | STXBP2 | XIAP |
| C8G | COPA | IFNGR1 | MALT1 | PTPRC | TAP1 | ZAP70 |
| C9 | CORO1A | IFNGR2 | MAP3K14 | RAB27A | TAP2 |  |
| CARD11 | CR2 | IGLL1 | MASP1 | RAC2 | TAPBP |  |
| CARD14 | CSF2RA | IKBKB | MASP2 | RAG1 | TAZ |  |
| CARD9 | CSF3R | ZBTB24 | MCM4 | RAG2 | TBK1 |  |
| CASP10 | CTLA4 | IKZF1 | MEFV | RBCK1 | TBX1 |  |
| CASP8 | CTPS1 | IL10 | MOGS | RFX5 | TCF3 |  |
| CCBE1 | CTSC | IL10RA | MRE11A | RFXANK | TCN2 |  |
| CD19 | CXCR4 | IL10RB | MS4A1 | RFXAP | TERT |  |
| CD247 | CYBA | IL12B | MSH6 | RHOH | THBD |  |
| CD27 | CYBB | IL12RB1 | MTHFD1 | RNASEH2A | TICAM1 |  |
| CD3D | DCLRE1B | IL17F | MVK | RNASEH2B | TINF2 |  |

**Supplemental Table 1. The list of the genes captured in the present study.**
